# In the pursuit of new social neurons. Neurogenesis and social behavior in mice: A systematic review

**DOI:** 10.3389/fcell.2022.1011657

**Published:** 2022-11-04

**Authors:** Lydia García-Gómez, Iker Castillo-Fernández, Ana Perez-Villalba

**Affiliations:** Laboratory of Animal Behavior Phenotype (L.A.B.P.), Department of Neuropsychology, Faculty of Psychology, Catholic University of Valencia, Valencia, Spain

**Keywords:** adult neurogenesis, hippocampus, social behavior, social memory, social stress, housing conditions, neural stem cells, parental behavior

## Abstract

Social behaviors have become more relevant to our understanding of the human nervous system because relationships with our peers may require and modulate adult neurogenesis. Here, we review the pieces of evidence we have to date for the divergence of social behaviors in mice by modulation of adult neurogenesis or if social behaviors and the social environment can drive a change in neurogenic processes. Social recognition and memory are deeply affected by antimitotic drugs and irradiation, while NSC transgenic mice may run with lower levels of social discrimination. Interestingly, social living conditions can create a big impact on neurogenesis. Social isolation and social defeat reduce the number of new neurons, while social dominance and enrichment of the social environment increase their number. These new “social neurons” trigger functional modifications with amazing transgenerational effects. All of these suggest that we are facing two bidirectional intertwined variables, and the great challenge now is to understand the cellular and genetic mechanisms that allow this relationship to be used therapeutically.

## 1 Introduction

Mice and humans are social animals, and both share a clear preference for social contact, group living, and a natural curiosity for novel social stimuli. This social behavior is associated not only with costs, like resource competition, but also with benefits, like cooperative breeding (Lee, 1994). Additionally, both mice and humans may also share the generation of new neurons in the adult brain (Kempermann et al., 2018). Adult neurogenesis is a complex biological process that occurs in two neurogenic niches of the adult mouse brain: the dentate gyrus (DG) of the hippocampus (Kempermann et al., 2004) and the subependymal zone (SEZ) of the lateral ventricles (Lois and Alvarez-Buylla, 1994). Sophisticated stages of proliferation, specification, and maturation are necessary for neuroblasts to be integrated in neural circuits. How frequent and feasible is this process in the adult human brain is still under interesting debate (Lucassen et al., 2020; Alvarez-Buylla et al., 2022).

In rodents, cognitive and emotional variables have been repeatedly correlated with variations in adult neurogenesis (Lazarini et al., 2009; Aimone et al., 2011). Extrinsic factors that regulate neurogenesis include enriched environment, voluntary exercise, and diet, skipping one of the most important variables: social behaviors. However, in recent years, an increasing number of articles include social behaviors in the list of parameters causing adult neurogenesis variations (Gobshtis et al., 2017; Pereira-Caixeta et al., 2017; Pereira-Caixeta et al., 2018; Lunardi et al., 2021), even though there is no clear agreement of their mutual influence.

Social behavior includes an extensive mosaic of different and complex behaviors, usually underrepresented or simplified but increasingly correlated with neurogenesis. Here, we review and analyze possible bidirectional effects: whether alterations in adult neurogenesis modulate social behavior and if social behaviors change neurogenesis.

## 2 Materials and methods

**FIGURE 1:**
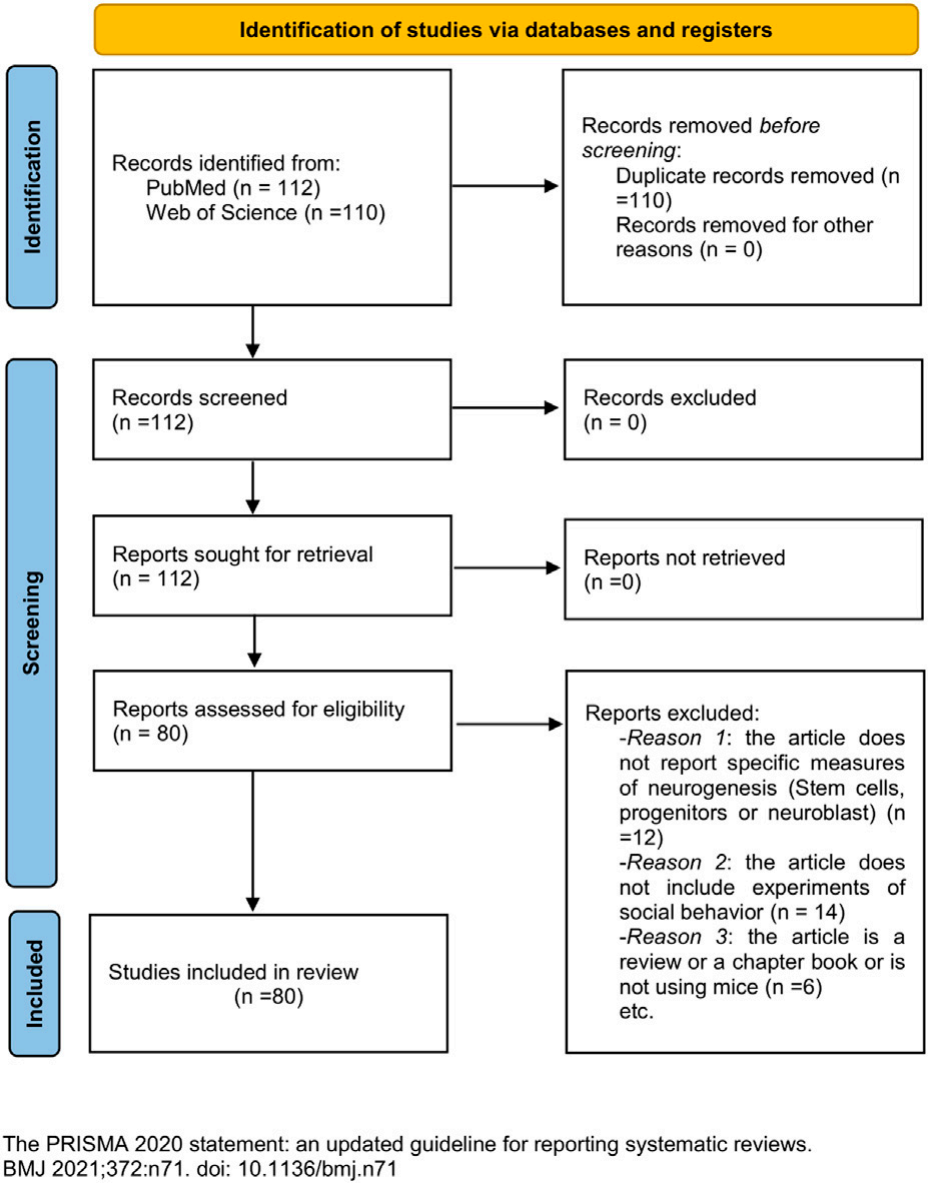
PRISMA flow diagram (2020 version).

We performed a systematic literature search in PubMed and WoS (open access) in accordance with the “Preferred Reporting Items for Systematic Reviews and Meta-Analyses” (PRISMA) guidelines (Page et al., 2021). The Boolean expression used for our search was as follows: “adult neurogenesis” AND “social behavior” AND “mice.” This search retrieved 112 articles. All the reviewed articles included randomization in the distribution of experimental subjects and were published in peer-reviewed journals without time boundaries. We defined inclusion/exclusion criteria. Inclusion criteria were as follows: 1) the article should report specific measures of neurogenesis, concerning stem cells, progenitor cells, or neuroblast; 2) the article should include experiments of social behavior (Supplementary Figure S1); and 3) social behavior could be a consequence or an inductor of neurogenesis changes. Exclusion criteria were as follows: a) reviews, b) book chapters, c) articles not using mice, and d) documents not written in English. Exclusion criteria affected six articles. For inclusion criteria accomplishment, 12 articles did not meet criteria (i) and 14 did not meet inclusion criteria (ii) or (iii) (PRISMA flow diagram shown in Figure 1). Therefore, we included a total of 80 articles in this systematic review (Supplementary Table S1). Additional references were used for contextualizing.

## 3 Discussion

### 3.1 Irradiation, chemical damage, and chemical recovery of neurogenesis and their impact on social behavior

**FIGURE 2:**
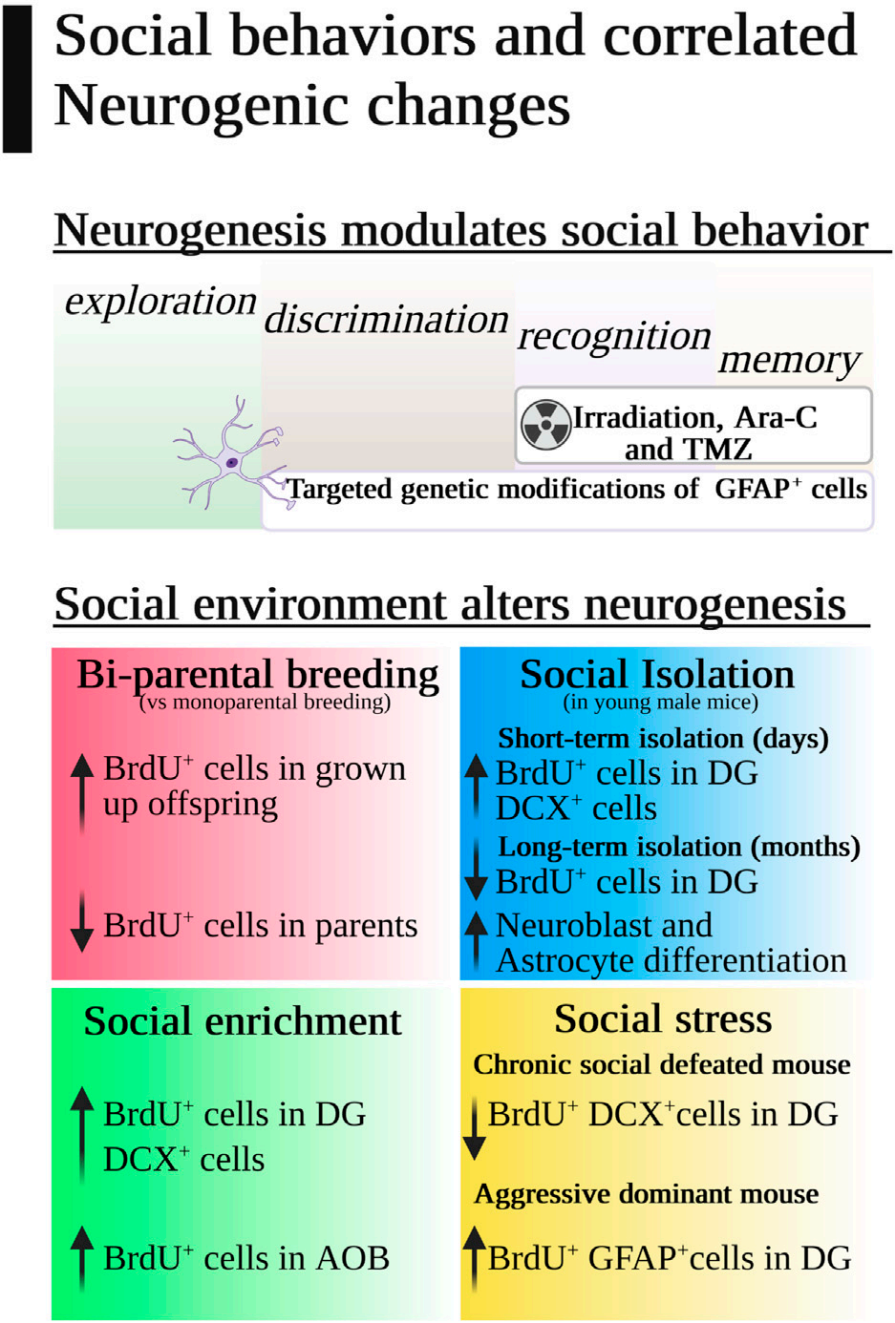
Social behaviors and correlated neurogenic changes.

The most popular chemical methods to disrupt adult neurogenesis are brain delivery of cytosine-β-D-arabinofuranoside (AraC) and injection of temozolomide (TMZ). Both antimitotic drugs have been correlated with disrupted social recognition and memory, but the highest correlation was achieved by gamma irradiation (Pereira-Caixeta et al., 2018). Whole brain irradiation in adult mice drastically reduces subgranular zone (SGZ) cells by 93%–96% and immature neurons from 40% to 60% in a dose-dependent fashion (Mizumatsu et al., 2003), and it has been used as an acute and effective neurogenesis inhibitor. It also affects the dendritic complexity of new neurons, which is directly associated with social memory disruption even in young mice (Newton et al., 2020), especially when radiation affects CA2, a hippocampal region known to be important for social memory (Hitti and Siegelbaum, 2014) (Figure 2).

But irradiation studies also demonstrated that adult neurogenesis is not a requirement for performing some social behaviors, such as maternal behavior, and for female mice to distinguish among pups. In contrast, adult olfactory neurogenesis is essential for normal social interaction, especially in male mice (Feierstein et al., 2010).

Additionally, chemical recovery of adult hippocampal neurogenesis (AHN) with psychoactive drugs has given rise to not fully understood results on social behavior. For example, memantine, the glutamate receptor antagonist, was associated with increased AHN (Maekawa et al., 2009) but not necessarily with an improvement in long-term social memory after previous 3 min of social contact, but it shows an association after reducing that time (Ishikawa et al., 2014).

### 3.2 Neurogenic changes induced by genetic mutations and the effect on social behavior

Unlike the local action of irradiation, many gene mutations are associated with a broad spectrum of abnormalities, like changes in development, neurotransmitters, neurogenesis, and behavior. We reproduce some examples here. The BTBR mouse model of autism deals with multisystem physiological and behavioral alterations, including social behavior deficits and a reduced number of bromodeoxyuridine (BrdU)^+^ cells, doublecortin (DCX)^+^ cells, nestin^+^ cells, and glial fibrillary acidic protein (GFAP)^+^ Sox2^+^ cells in the hippocampus, but researchers can normalize their neurogenesis and social behavior by transplanting human amniotic epithelial cells (Zhang R. et al., 2019). In contrast, another model of autism, PTEN (phosphatase and tensin homolog on chromosome 10) mutant mice, showed deficits in social exploration despite having increased Ki67^+^ cells in the DG (Amiri et al., 2012) in addition to developmental and electrophysiological alterations. In the same line of indirect relationships, knock-out mice of the cell adhesion molecule neuroplastin-65 showed anxious-like and depressive-like behavior as well as more BrdU^+^ cells in the DG and SEZ (Li et al., 2019). Here, more NSCs are associated with not only increased social interaction but also downregulation of serotonin affecting neurogenic niches and the limbic system. The same lack of specificity was described with poly (ADP-ribose) polymerase-1 (PARP-1, a multi-functional nuclear enzyme that regulates DNA repair). PARP-1 knock-out mice showed not only a high impact on NSC survival, DCX^+^ cells, and low neuronal differentiation that is associated with diminished social interaction (Hong et al., 2019) but also with symptoms described as “schizophrenia-like behavioral deficits” that include hyperactivity and anxiety. This is a similar profile to other “schizophrenia-like” DISC1 and BRINP1 mouse models. The mutant expression of DISC1 produced more anxious and depression-like behavior and reduced social exploration in the social chamber test as well as less proliferation of neural progenitors and dendritic maturation in the DG (Terrillion et al., 2017). Reduced neurogenesis is presented with reduced sociability (among other alterations). In BRINP1 KO mice, the lack of this cell cycle suppressor resulted in increased cell division and neurogenesis (Kobayashi et al., 2014). They also presented hyperactivity and slight impulsive response. Then, they showed a reduced duration of contacts in the social interaction test but a radical increase in total distance travelled suggesting that hyperactivity may affect sociability by reducing the length of contact despite preserving social curiosity (with an equal number of contacts). Once again, this is a scenario of multi-functional alterations.

These broad-spectrum variables are presented in articles that related social exploration and neurogenesis triggered by genetic mutations that affected numerous systems (Lehmann et al., 2013; Guha et al., 2014; Garrett et al., 2018; Alen et al., 2019; Zhang et al., 2019; Amar et al., 2021; Velloso et al., 2022), types of cells and proteins (Jenniches et al., 2016; Zhang et al., 2016; Salminen et al., 2017; Alén et al., 2019; Zhang et al., 2020; Nacer et al., 2021) plus multisystem drugs (Poletaeva et al., 2012; Osborn et al., 2013; Subbanna and Basavarajappa 2014; Chiu et al., 2015; Di Nuzzo et al., 2015; Castilla-Ortega et al., 2016; Fu et al., 2018; Jiang et al., 2020; Kim et al., 2020), or in addition to electrophysiological changes in neurons (Alachkar et al., 2013). Therefore, the extensive effects of genetic mutations make it difficult to deduce a direct effect on social behavior (Bagnall-Moreau et al., 2020).

Instead, when genetic modifications were specifically guided with inducible and reversible genetic models to reduce neurogenesis, social discrimination between a familiar and a new mouse could be correlated with the number of DCX in the DG (Garrett et al., 2015) without altering other behaviors. Pharmacological reduction of AHN with transgenic mice expressing herpes-simplex virus-TK (thymidine kinase) under the GFAP promoter affected social memory (Cope et al., 2020).

Transgenic GFAP-TK-GFP mice injected with ganciclovir in the lateral ventricle showed a massive elimination of DCX^+^ cells in the MOB (main olfactory bulb) and DG without affecting astrocyte lineage or microglia (Wei et al., 2011). Curiously, when the investigators eliminated neurogenesis (DCX^+^ cells) in adolescence, there were no changes in memory and anxiety tests, but social exploration was severely affected. However, when the same experiment was performed on adult mice, there was no effect on social behavior. Normal socialization requires neurogenesis during adolescence, but once socialization is established, neurogenesis was no longer required. It would be interesting to study if this effect can be reversed over time.

Finally, social recognition and hence social memory in mice depend on the olfactory system, which receive neuroblasts through the rostral migratory stream from the SEZ (Lledó and Valley, 2016). Accordingly, the reduction of DCX in the MOB has a functional correlation with social memory (Garrett et al., 2018) even with high levels of proliferating cell nuclear antigen (PCNA) in the SEZ, suggesting that social memory formation needs intact olfactory and hippocampal neurogenesis.

### 3.3 Voluntary exercise as a neurogenic booster with social behavior modulation

Voluntary exercise can increase AHN (van Praag et al., 1999), and now we know several social variables that could modulate this classic effect. When young male mice are isolated and stressed, exercise increases short-term (Kannangara et al., 2009) and long-term (Hueston et al., 2017) BrdU^+^-retaining cells but only if they are left isolated and the effect is not reproduced if they are grouped, supporting the idea that social housing can buffer the effects of stress. Actually, the more severe and stressful the conditions in which neurogenesis is reduced, the greater the effect of anxiolytic variables, such as physical exercise and social contact. Moreover, social isolation during adolescence prevented exercise-induced neurogenesis in the ventral hippocampus with similar numbers in long-term BrdU^+^ and NeuN^+^ cells with or without voluntary wheel running (Kozareva et al., 2018). Another study found that environmental and social complexity did not directly regulate running-induced neurogenesis (Grégoire et al., 2014). Here, isolated mice with voluntary running had more Ki67^+^ and long-term BrdU^+^-retaining cells in the DG than isolated mice living with a locked running wheel, but not statistically different than socially enhanced conditions. This would suggest that exercise *per se* is not compensating neurogenesis by substituting the effect of isolation and poor environments.

### 3.4 Social behavior as a modulator of neurogenic changes

#### 3.4.1 How social living conditions affect neurogenesis

Usually, inbred mice are housed to resemble social co-habitant conditions in nature, with some relevant exceptions with consequences in adult neurogenesis and social behavior (Figure 2). For instance, mice pups are weaned 21 days after birth, a period of time in which pups are very sensitive to the social environment. Single-parental conditions are the standard condition for breeding mice pups because pregnant mothers are separated from males and take care of the pups alone. However, bi-parental housing (mother and father or mother and another female) increases BrdU^+^ cells in the DG of male mice pups when they become adults in comparison with single-parental housing, and this effect improves context fear conditioning and is transmitted to the next generation (Mak et al., 2013). Curiously, offspring females with bi-parental housing did not change DG neurogenesis, but they increased oligodendrogenesis in the corpus callosum and their social exploration in the social chamber test, showing that female mice AHN is more resilient to early life social stressors than male mice AHN. Unfortunately, the neurogenic effect for progenitors was the opposite as parenting decreases BrdU^+^ cells in the DG in male and female parents compared to controls (Glasper et al., 2011).

One of the most emotional and cognitive impacting housing conditions for mice is social isolation (Figure 2), especially for male mice (Guarnieri et al., 2020) although no clear effect on hippocampus cell proliferation has been demonstrated *per se* (Monteiro et al., 2014; Guarnieri et al., 2020). Young males isolated for a short term could have a greater number of BrdU^+^ cells in the DG after only 4 days of isolation, but surprisingly this difference was not found in females, and it disappeared 20 days later (Ruscio et al., 2015). Long-term isolation studies found a moderate influence on neurogenesis through an increase in neuroblast and astrocyte differentiation (Du Preez et al., 2020; Du Preez et al., 2021) that can be reversed with physical exercise (Hueston et al., 2017). This could mean that exercise, in comparison with social isolation, did not change the number of BrdU^+^ cells in isolated adult mice but could buffer the neuronal and astrocyte differentiation effect. Prolonged social isolation resulted in more undifferentiated NSCs and fewer DCX^+^ cells in the DG than in standard grouped housing (Dranovsky et al., 2011), proposing a time-dependent influence of social isolation on the fate and lineage of NSCs.

Furthermore, when social isolation was applied in mice pups, more important changes occur in NSCs. Some authors report an increase in proliferation in the SEZ with more BrdU^+^ cells, more neurospheres in the primary culture, and more BrdU^+^ GFAP^+^ cells in the SEZ of maternal and socially deprived mice (Daun et al., 2020), which could raise not only neurogenesis but also NSC exhaustion. However, others found lower levels of BrdU in the ventral DG of maternal deprived mice (O’Leary et al., 2014) or fewer NeuN^+^ cells in CA3 (Reshetnikov et al., 2020), showing that both neurogenic niches respond differently to social isolation. Nevertheless, this is not necessarily a contradictory result as new neurons accomplish different functions in the two neurogenic niches. Functions like olfactory exploration and memory consolidation could be stimulated or deactivated simultaneously by social isolation.

There are myriad molecular mechanisms that affect NSCs and their transition from quiescent, primed-for-activation, and activated cells. For example, inflammatory signals like interleukin 17A serum levels in a murine model of posttraumatic stress disorder modulate DCX and Ki67+cells in the DG (Willinger and Turgeman 2022) or extracellular and adhesion molecules (reviewed in Morante-Redolat and Porlan, 2019) can promote alertness and activation of NSCs in response to indicators that affect the entire organism. Tumor necrosis factor-α (TNF-α), a pro-inflammatory cytokine, induces multiple effects in the nervous system through two main receptors: TNF-R1 and TNF-R2, which play very different functions in neurogenic niches (Belenguer et al., 2021). Removing the TNF receptor results in different social behaviors. While TNFR1^−/−^ mice seemed perfectly normal in social exploration, recognition, and memory, TNFR2^−/−^ mice showed lower social exploration and social memory (Camara et al., 2013).

Threats to the immune system are linked with transgenerational effects on social behavior. Social novelty exploration is diminished in adult mice when the mother had viral immune activation during pregnancy. Consequently, a recreation of this condition in mice is counterbalanced with vaccination (Wu et al., 2018). Other authors found that social isolation primed the immune system of isolated mice for infection allowing them to clear bacterial threats more effectively than grouped mice (Hamilton et al., 2022). Thus, housing conditions could be a key variable to understand fluctuations in NSC activity through immune system modification.

Similarly, stimulating social contact has been presented as a neurogenic trigger in APP/PS1 mice, an established model for Alzheimer’s disease with progressive spatial memory failure. Notably, these mice showed improved performance when they are co-housed with healthy young mice (Hsiao et al., 2014). This benefit in cognitive performance through the BDNF-TrkB signaling pathway was activated in aged APP/PS1 mice but we do not know if this partnership is beneficial for younger mice or if this effect is seemingly happening in physiological aging.

#### 3.4.2 How social enrichment affects neurogenesis

Adult neurogenesis in rodents can be enhanced by transferring mice from standard laboratory housing to a more complex and stimulating environment (Kempermann et al., 1997; Kempermann et al., 1998). One main component of this environmental enrichment was social stimuli (Figure 2). Social enrichment can trigger hippocampal DCX division by itself in the same way as that in contact with objects and running wheels (Moreno-Jiménez et al., 2019). After tamoxifen neurogenesis depletion in the hippocampus, social environmental enrichment was able to generate the highest level of new neurons compared to standard or isolated housing (Dranovsky et al., 2011). Moreover, female mice in an enriched environment with male pheromones would need adult neurogenesis to develop mate preferences and equivalently, environmental pheromone cues induce olfactory and DG neurogenesis (Mak et al., 2007), indicating that socially rich environments promote neurogenesis (Sakalem et al., 2017). In the olfactory system, social behaviors relying on the activation of the VNS regulate adult neurogenesis in the mouse AOB. For example, male mice presented more BrdU^+^ cells when they were intruders in a resident cage and female mice had higher numbers of BrdU^+^ cells when they were in contact with male urine, although both sexes decreased new neurons in the AOB with aging (Nunez-Parra et al., 2011). Finally, social enrichment could be associated with sexual contact that activates neurogenesis and counterbalances the effect of chronic stress (Kim et al., 2013).

Likewise, social and environmental enrichment in young mice promotes increased social exploration and interaction with a novel mouse or object, despite this being not necessarily associated with an increase in AHN (Silva et al., 2011; Buschert et al., 2016). Curiously, when environmental enrichment is used to get a recovery from previous social stress conditions, the stimulation of adult neurogenesis is required (Schloesser et al., 2010). Therefore, social enrichment could be a therapeutic resource as long as adult neurogenesis can be stimulated.

Excessive social contact in crowded housing did not induce BrdU changes in the hippocampus at short or long intervals (Ago et al., 2014), and there are no immediate consequences on anxious behavior, highlighting high sociality in mice.

#### 3.4.3 How social stress affects neurogenesis

The social role of a mouse in a group is based on a relatively stable hierarchy with acute and mild social pressure and aggression to keep the individuals in the social rank (Jennings et al., 1998). Consequently, any alteration in this dynamic system induces an extra dose of social stress. This stressor can be recreated with repeated social defeat or with an unstable social hierarchy. In repeated social defeat, the experimental mouse is exposed to recurrent social aggressions from a dominant mouse. This procedure induces avoidance to the aggressor, anxious behavior (Mouri et al., 2018), long-term disruption in AHN (Mitra et al., 2006; Chen et al., 2015; McKim et al., 2016), inflammatory local response (Ito et al., 2017; Shen et al., 2022), and decreased social interaction that can be recovered with antidepressants (Wu et al., 2017) and glucocorticoids (Lehmann et al., 2013).

One month after social defeat, adult male mice reduced the numbers of BrdU^+^ DCX^+^ cells (McKim et al., 2016) or their BrdU^+^ Neu-N^+^ cells (Chen et al., 2015; Jiang et al., 2017), suggesting that social stress affects neural progenitor cell differentiation (Figure 2). Again, higher differences can be observed when juvenile mice were used in which chronic social defeat was associated not only with lower neurogenesis but also with diminished Ki67^+^cells (Mouri et al., 2018). In contrast, some reliance to social stress induced by defeated experience has been described in adult and adolescent GFAP-TK heterozygous mice with reduced neurogenesis (Lehmann et al., 2013; Kirshenbaum et al., 2014). Curiously, DCX knock-out mice reduced their aggressiveness in the resident-intruder paradigm (Germain et al., 2013), and hypertrophic *Pten* ^LoxP^Nestin-CreER^T2^ mice have more Ki67 in the DG without affecting spatial hippocampal functioning but with deficits in social interaction (Amiri et al., 2012). Different results appeared when we consider mouse strain and total BrdU^+^ cells in the DG. C57BL/6 × 129/Sv mice showed lower social interaction after repeated social stress together with higher number of BrdU^+^ cells (McAllister et al., 2020). C57BL/6N mice showed lower social interaction after repeated social stress but without changes in BrdU^+^ cell numbers (Ishikawa et al., 2019), and OF1 mice showed more aggressive behavior after repeated social stress with fewer BrdU^+^ cells (Ferragud et al., 2010). Therefore, the mouse strain could affect social stress output, neurogenesis, or both.

An alternative protocol for social stress in the setting of social hierarchy is called social confrontation stress. This is a repeated aggressive experience to establish social dominance, and it classifies mice between “winners” (more offensive behavior) and “losers” (more defensive behavior). Interestingly, winners had a higher number of BrdU^+^ cells, BrdU^+^GFAP^+^ cells, and DCX^+^ cells and amplifying neural progenitor cells (Smagin et al., 2015) but only when this social dominance was established through explicit aggressive experience. Non-aggressive social dominance in the social tube test was not associated with changes in AHN (Pallé et al., 2019). Thus, developing explicit social dominance and not just social status enhances AHN.

Unstable housing conditions are another chronic social stress paradigm that increases BrdU^+^ cells in the DG (Buschert et al., 2013), but this is not associated with increased neurogenesis (Yohn et al., 2019). Actually, mice with extra copies of S100β are more sensitive to this housing, which has been linked to human major depression (Buschert et al., 2013). This implies that unstable housing could be a mild stressor in comparison to chronic social defeat that gets a bigger impact in neurogenic niches.

Finally, adult neurogenesis can be modulated by the social environment, and also new “social neurons” can transform our social behavior (Figure 2). Now, we would need to understand how we can use this reciprocal relationship for therapeutic purposes.

## Supplementary material

SUPPLEMENTARY FIGURE S1 Social behavior tests used in the articles of this review. **(A)** *Three-chamber test (or social chamber test)* in which mice are placed in three open compartment spaces and find the possibility to explore objects and/or other mice (Rein et al., 2020 for protocol) or **(B)** *social interaction test*, in an open-field arena mice, interact for few minutes. Social hierarchy and dominance are evaluated using the **(C)** *social tube test* (Fan et al., 2019 for protocol). Social stress can be observed with the *resident-intruder paradigm and repeated social defeat* **(D)**. The resident acts as an aggressor, and the intruder shows defensive and avoiding behavior.

SUPPLEMENTARY TABLE S1 References reviewed in this systematic review according to the PRISMA flow diagram. Title, authors, year of publication, and DOI are reported for every article.

## Abbreviations

AHN, adult hippocampal neurogenesis; AOB, accessory olfactory bulb; BrdU, 5-bromo-2-deoxyuridine; DCX, doublecortin; DG, dentate gyrus; GFAP, glial fibrillary acidic protein; MOB, main olfactory bulb; NSCs, neural stem cells; SEZ, subependymal zone; SGZ, subgranular zone; VNS, vomeronasal system.
