# Supplementary figures and images for: In the pursuit of new social neurons. Neurogenesis and social behavior in mice: A systematic review
Source: Front Cell Dev Biol. 2022 Nov 4;10:1011657. doi: 10.3389/fcell.2022.1011657 (PMC9672322; doi:10.3389/fcell.2022.1011657)

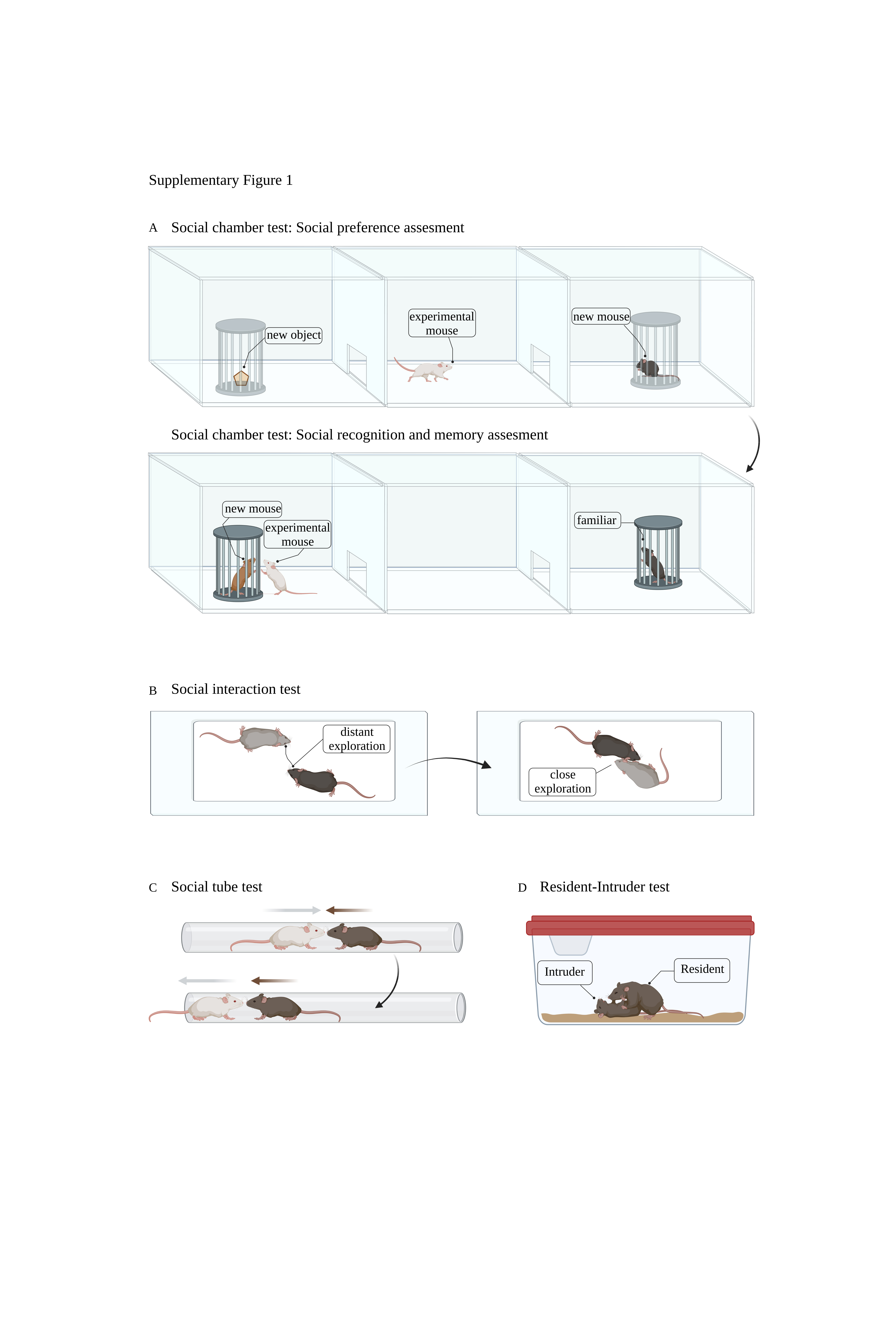

Supplement: Supplementary file 1 [file Image1.JPEG]
